# Convolution Comparison Pattern: An Efficient Local Image Descriptor for Fingerprint Liveness Detection

**DOI:** 10.1371/journal.pone.0148552

**Published:** 2016-02-04

**Authors:** Carsten Gottschlich

**Affiliations:** Institute for Mathematical Stochastics, University of Göttingen, Goldschmidtstr. 7, 37077 Göttingen, Germany; Nanjing University of Aeronautic and Astronautics, CHINA

## Abstract

We present a new type of local image descriptor which yields binary patterns from small image patches. For the application to fingerprint liveness detection, we achieve rotation invariant image patches by taking the fingerprint segmentation and orientation field into account. We compute the discrete cosine transform (DCT) for these rotation invariant patches and attain binary patterns by comparing pairs of two DCT coefficients. These patterns are summarized into one or more histograms per image. Each histogram comprises the relative frequencies of pattern occurrences. Multiple histograms are concatenated and the resulting feature vector is used for image classification. We name this novel type of descriptor convolution comparison pattern (CCP). Experimental results show the usefulness of the proposed CCP descriptor for fingerprint liveness detection. CCP outperforms other local image descriptors such as LBP, LPQ and WLD on the LivDet 2013 benchmark. The CCP descriptor is a general type of local image descriptor which we expect to prove useful in areas beyond fingerprint liveness detection such as biological and medical image processing, texture recognition, face recognition and iris recognition, liveness detection for face and iris images, and machine vision for surface inspection and material classification.

## 1 Introduction

Local image descriptors comprise and encode pieces of information in a local neighborhood ranging from only few pixels to small image patches. These descriptors are very useful in a multitude of applications in pattern recognition and computer vision [1], like e.g. texture recognition [2, 3], optical character recognition, biological or medical image analysis (e.g. virus recognition [4]), machine inspection of surfaces [5] or biometric recognition. Examples of local image descriptors are local binary patterns (LBP) [6], gray-level co-occurrence matrices (GLCM) [7], Gabor filters (GFs) [8], scale-invariant feature transform (SIFT) [9], Weber local descriptor (WLD) [10], intensity-domain spin images [11] and rotation-invariant feature transform (RIFT) [11]. Many further descriptors are discussed in [1, 12]. Often, these local descriptors are summarized by a single histogram per image (e.g. LBP or WLD) which can be used as a feature vector for image classification (e.g. by support vector machines [13]), signatures [11] are classified by the earth mover’s distance [14], or joint probability density functions of patterns are described by Gaussian mixture models [15].

In this manuscript, we introduce a new local image descriptor based on the discrete cosine transform (DCT) which obtains a binary pattern from a comparison of DCT coefficients. We denote this novel descriptor as convolution comparison pattern (CCP) and we study the application of the proposed CCP descriptor for fingerprint liveness detection. The discrete cosine transform has been introduced in 1974 [16]. An in-depth discussion of the DCT and many additional references can be found in [17]. The DCT is well-known for its use in image compression (JPEG) and video compression (MPEG, Daala, and Theora) [18]. In the context of fingerprint recognition, the DCT has been considered for fingerprint matching [19], for fingerprint image enhancement [20], for fingerprint image compression [21] and for estimating the quality of fingerprint images captured by smartphone cameras [22].

### 1.1 Fingerprint Liveness Detection

Hundreds of millions of people use fingerprint recognition in their daily life, especially for unlocking their smartphone, and increasingly, for authorizing financial transactions. As a consequence, attacking fingerprint recognition systems is becoming more and more attractive for criminals. Two important types of attacks are resembling impostor attacks [23] and spoof attacks [24, 25]. Spoofs are fake fingers produced from material such as gelatin, wood glue or silicone. These artificial fingers intend to fake the presence of real finger to a sensor. Several scenarios are conceivable how spoof fingers can be created. For example, a fingerprint can be lifted from a glass or another object previously touched. The image can be automatically enhanced using typical fingerprint preprocessing steps [26] like segmentation, orientation field estimation, ridge frequency estimation [8] and fingerprint enhancement [8, 27]. Finally, the enhanced image can be printed to obtain a mold [24]. Another possibility is the reconstruction of a fingerprint image from a stolen minutiae template. First, the segmentation and orientation field is reconstructed [28]. Next, image reconstruction can be achieved e.g. using amplitude- and frequency-modulated (AM-FM) functions [29]. A survey of fingerprint reconstruction methods is given in [30].

Software-based liveness detection is a very suitable countermeasure against spoof attacks. An acquired fingerprint image is not only used for fingerprint verification (e.g. unlocking a smartphone) or fingerprint identification (e.g. watch list search at border control), but the same image is classified by a software module as ‘live’ or ‘spoof’. So called static methods perform this classification based on a single image, whereas so called dynamic methods require a series of images as input. Approaches for liveness detection employ general image descriptors which are used e.g. in texture recognition and other areas of application, as well as fingerprint specific features like the ridge frequency [31] or finger pores [32]. Currently, state-of-the-art performance in software-based liveness detection is achieved by local image descriptors and by deep convolutional networks [33, 34]. An overview of approaches which apply local image descriptors for liveness detection is given in [35]. However, drawbacks of deep convolutional networks are the computational complexity and runtime, and recently it has been shown that deep neural networks are vulnerable to attacks with artificial images [36]. More references to software-based fingerprint liveness detection methods can be found in Section 3 and in [24, 25, 37].

## 2 Comparison of DCT Coefficients

**Fig 1:**
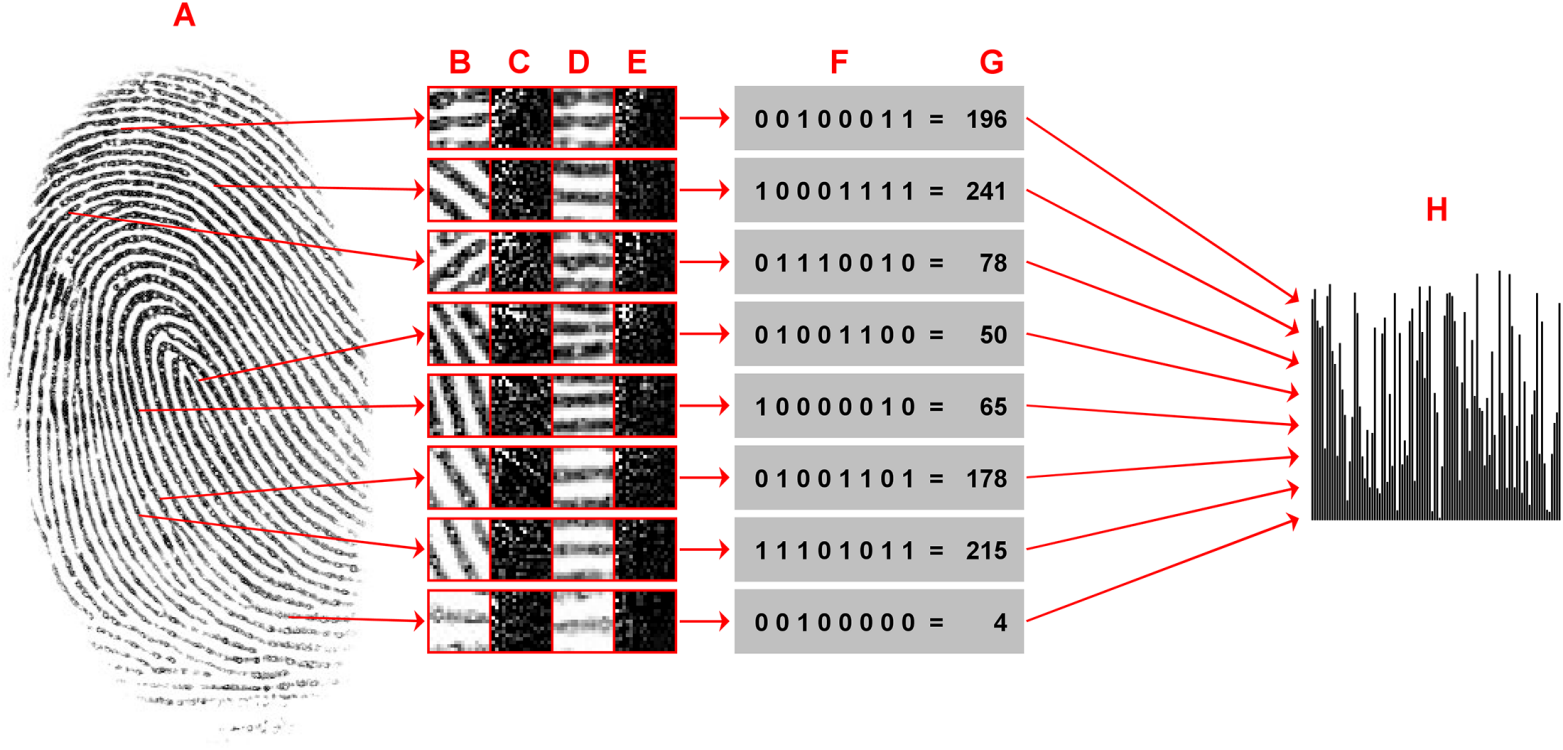
Overview over the proposed feature vector computation. Every foreground pixel of a fingerprint (A) is considered as the center of a small image patch. Instead of using the original patches (B) and their DCT coefficients (C), we take the local orientation into account to obtain rotation invariant patches (D) and their DCT coefficients (E). We compute the binary pattern (F) by comparing selected pairs of two DCT coefficients from (E), see Eq (2). The pattern (F) is converted intoa bin number (G), see Eq (3). A histogram (H) summarizes the relative frequency of occurrence of all local patterns for an image. (For illustrative purposes only, patch sizes are here 17×17 pixels, and coefficients with index = 1 are set to zero in (C) and (E), and (F-H) show example descriptors.)

**Fig 2:**
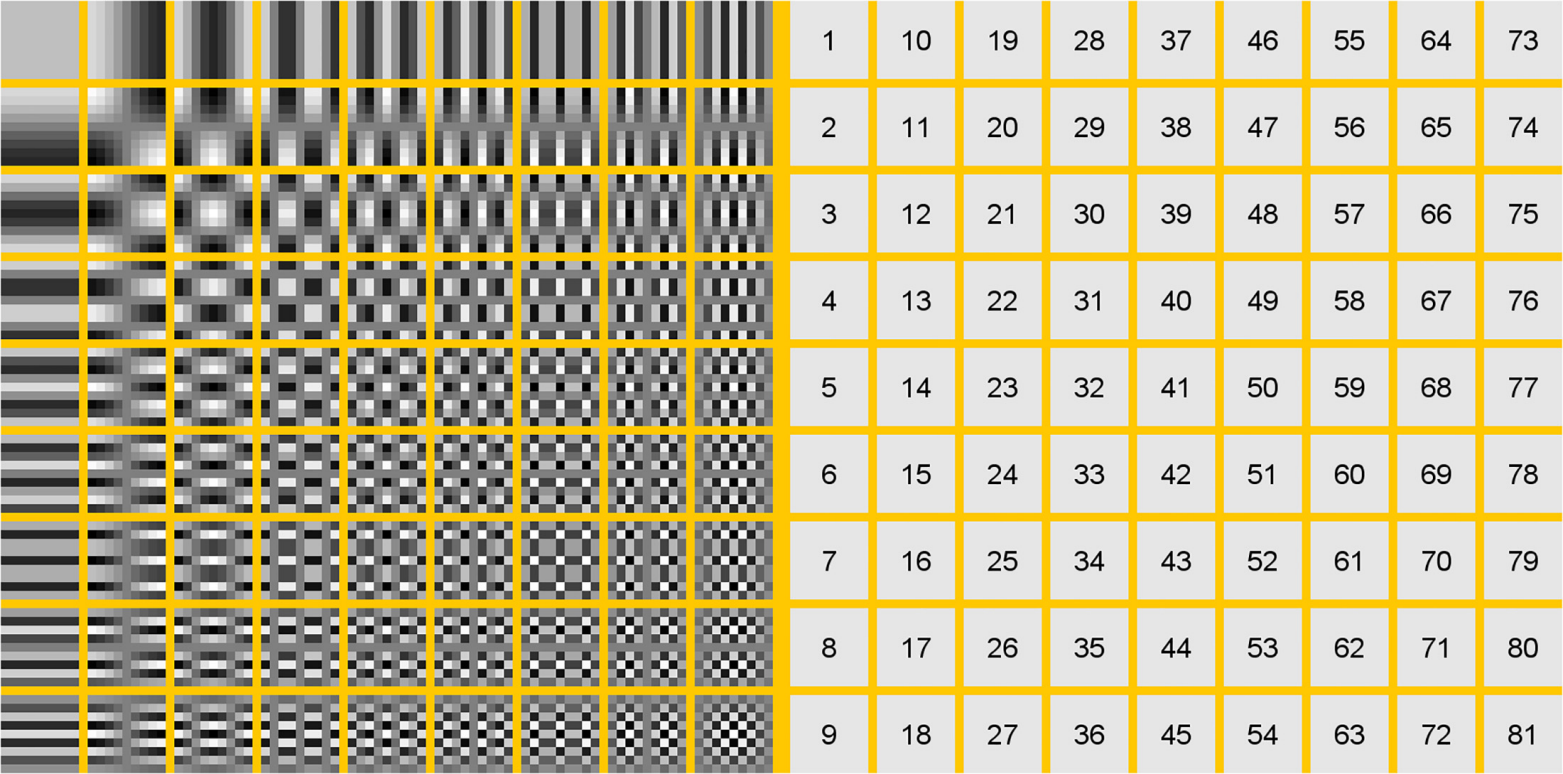
Visualization of DCT coefficients for *n* = 9 (left) and corresponding index numbers (right). Negative values are depicted in black or dark gray and positive coefficients are shown in white or light gray.

An overview over the CCP feature computation is given in Fig 1 and the DCT basic elements for *n* = 9 (patch width in pixels) are visualized in Fig 2. Here, fingerprint images are used as examples of image classification by CCP. The following framework is applicable to other types of images, like e.g. microscopy images in biomedical applications or texture recognition:

1. Preprocessing
2. Learning stage (Training)
3. Classification (Test)

### 2.1 Preprocessing

**Fig 3:**
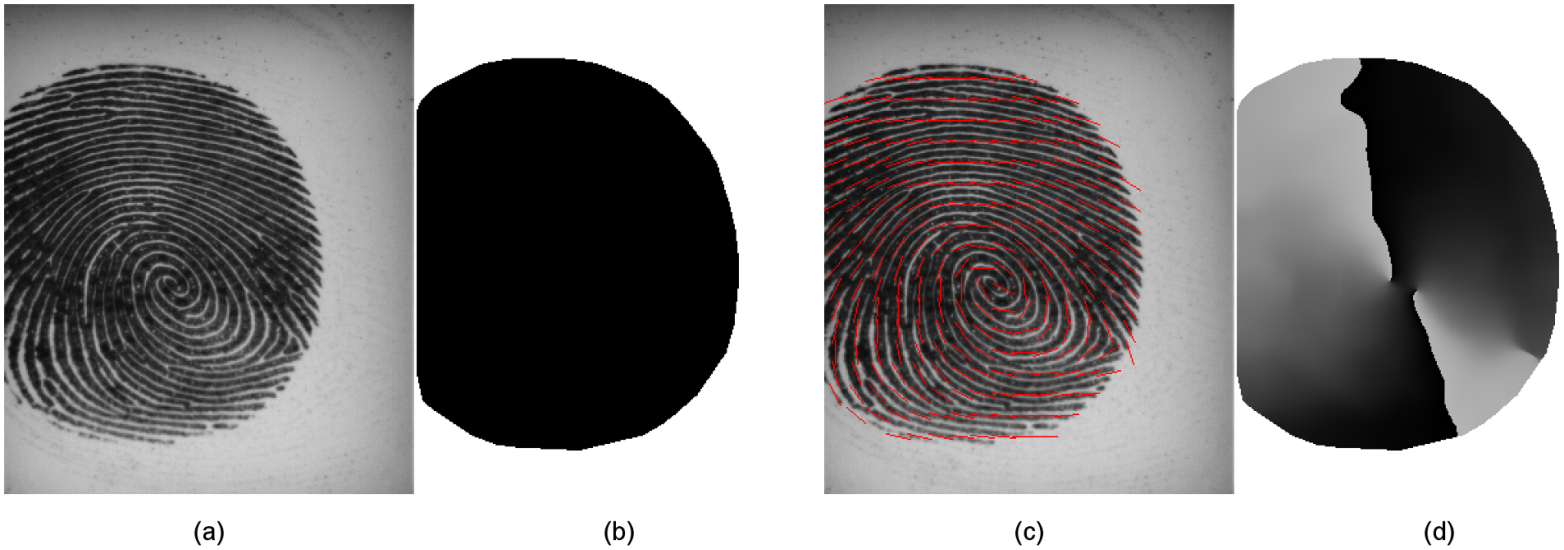
The preprocessing steps for an input fingerprint image (a) are segmentation (b) and orientation field (OF) estimation (c,d). Foreground pixels in (b) are shown in black, background pixels in white. The OF is visualized in (c) by red lines for every 16th pixel. In (d), orientations in degrees are encoded by gray values between 0 and 179, where 0 corresponds to x-axis and angles increase clock-wise.

The two preprocessing steps are image segmentation and orientation field estimation (see Fig 3 for an illustration). We perform fingerprint segmentation by the FDB method [38]. A Matlab implementation of the FDB method is available for download at http://dx.doi.org/10.6084/m9.figshare.1294210. The goal of this step is to consider only those regions of an image which contain relevant information and to exclude the background area. Segmentation should also be performed for other applications like medical image classification, if some parts of the image are irrelevant for the classification task.

The second step is estimating an orientation field by averaging squared image gradients [27, 39]. We use the Sobel operator [40] for approximating gradients. The window size is set to 33×33 pixels and gradients are weighted by a Gaussian with *σ*^2^ = 10. Fingerprints are oriented patterns with one dominant orientation [41] at each location (with the exception of singular points, e.g. a delta is a location where three different orientations meet). The estimated local orienation is used to compute a rotation invariant image patch (see column D in Fig 1) by bilinear interpolation [40].

### 2.2 Learning stage

The proposed descriptor computes a binary pattern of *b* bits by comparing pairs of DCT coefficients. Every of the *b* comparisons involves two coefficients and the output is either a ‘0’ or a ‘1’. In order to select those comparisons which have the greatest discriminative power, we start by computing coefficient statistics, e.g. for *n* = 9, there are *n*^2^ = 81 DCT coefficients (see Fig 2), hence there are *n*^2^⋅(*n*^2^−1) = 6480 possible comparisons of two DCT coefficients, if we restrict ourselves in Eq (2) to a “greater than” (“>”) comparison.

We are interested in a small number, e.g. the 8 or 24 ‘best’ comparisons.

For illustrative purposes, let us consider the Biometrika database of LivDet 2013 [42] which consists of *t* = 2000 images for training and 2000 images for testing. Each set contains *t*_live_ = 1000 live and *t*_spoof_ = 1000 spoof samples. For each training image, we select *p* = 2000 pixel locations independently and uniformly at random from the foreground. For these *t*⋅*p* = 4,000,000 rotation invariant image patches of size *n*×*n* pixels, we compute DCT coefficients. Let be *d*_*i*_ the *i*-th DCT coefficient (*i* = 1, …, 81 in our example) and *a*_*i*_ = |*d*_*i*_| the absolute value of the respective coefficient. Let be $\bar{a_{i}}$ the mean value of all *a*_*i*_, i.e. $\bar{a_{i}}=\frac{1}{t\cdot p}\sum_{t,p}a_{i}$, $V_{i}=\frac{1}{t\cdot p}\sum_{t,p}{\left(a_{i}-\bar{a_{i}}\right)}^{2}$ the variance and $\sigma_{i}=\sqrt{V_{i}}$ the standard deviation. Next, we compute normalized coefficients $c_{i}=\frac{\left(a_{i}-\bar{a_{i}}\right)}{\sigma_{i}}$.

After this preparation, we compute the following statistic for both classes (‘live’ and ‘spoof’) in the training set separately:

$$\begin{array}{c} f\left(i,j,\delta \right)=\left\{\begin{array}{cc} 1 & \text{if}\left(c_{i}+\delta \right)>c_{j}\\ 0 & \text{otherwise} \end{array}\right.\;i\neq j,\delta \in R. \end{array}$$

Now, we select *i*, *j* and *δ* according to the following criterion:

$$\begin{array}{c} \max_{i,j,\delta }\left|\frac{1}{t_{\text{live}}\cdot p}\cdot \sum_{t_{\text{live}}}\sum_{p}f_{\text{live}}\left(i,j,\delta \right)-\frac{1}{t_{\text{spoof}}\cdot p}\cdot \sum_{t_{\text{spoof}}}\sum_{p}f_{\text{spoof}}\left(i,j,\delta \right)\right|. \end{array}$$ (1)

In our experiments, we let *δ* vary in the range from −1 to 1 in steps of size 0.02, and we find the best *i*, *j* and *δ* by exhaustive search. By computing these coefficient statistics, we learn for the Biometrika database that for *i* = 1, *j* = 11 and *δ* = −0.48, there is a difference of relative occurrence frequencies of 23.6% between the two classes ‘live’ and ‘spoof’. The difference of relative occurrence frequencies for *i* = 11, *j* = 72 and *δ* = −0.4 amounts to 21.1% (see Fig 2 for a visualization of the index numbers *i*, *j* and the corresponding DCT basis elements). To obtain a pattern with *b* bits, we choose the first *x* = 0, …, *b*−1 comparisons with *i*_*x*_, *j*_*x*_ and *δ*_*x*_ according to Eq (1) such that each combination of *i* and *j* is unique.

$$\begin{array}{c} q\left(i_{x},j_{x},\delta_{x}\right)=\left\{\begin{array}{cc} 1 & \text{if}\left(c_{i_{x}}+\delta_{x}\right)>c_{j_{x}}\\ 0 & \text{otherwise} \end{array}\right. \end{array}$$ (2)

$$\begin{array}{c} y=\sum_{x=0}^{b-1}\left(2^{x}\cdot q\left(i_{x},j_{x},\delta_{x}\right)\right) \end{array}$$ (3)

Hence, for *b* comparisons, the dimension of the resulting feature vector is 2*^b^*. In the next section and in Table 1, we report results for *b* = 8 which leads to a histogram with 2^8^ = 256. Additionally, we consider a feature vector obtained from the concatenation of four histograms with *b* = 6. The dimension of the vector for this choice is 4⋅2^6^ = 4⋅64 = 256 is the same as before. For *b* = 8, we model the joint distribution of 8 comparisons. In the alternative case, we model four times the joint distribution of 6 comparisons.

**Table 1:** Comparison of liveness detection methods in terms of accuracy in percent for LivDet 2013 databases [42]. Further results can be found in Table 7 of [42]. The description of CCP *n*×*b* bit can be found in Section 3.1.

	Biometrika	Italdata	Crossmatch	Average
WLD	93.9	90.9	50.0	78.3
LBP	98.9	96.3	49.5	81.6
MLPQ	98.2	97.9	50.2	82.1
WLD+MLPQ	99.0	97.7	44.5	80.4
LBP+MLPQ	98.6	97.7	45.4	80.6
UniNap1 [42]	95.3	96.5	68.8	86.9
Pore Analysis [47]	97.8	99.0	65.1	87.3
HIG [37]	96.1	98.3	71.2	88.5
Proposed convolution comparison pattern (CCP)				
CCP 1 × 8 bit	96.9	98.4	76.8	90.7
CCP 4 × 6 bit	97.9	98.5	82.5	**93.0**
CCP 2 × 8 bit	97.2	99.3	82.5	**93.0**
CCP 8 × 6 bit	98.0	99.3	76.5	91.3

Next, each individual histogram is normalized. We divide by the number of patches in the image to obtain the relative frequency of occurrence. In doing so, we achieve invariance with regard to the size of the foreground area. This area varies depending on the amount of surface area of the finger or spoof that touches the sensor and the CCP descriptor should not be influenced by this factor.

Finally, we compute the CCP feature vectors for all images of the training set and we train a machine learning algorithm for prediction or classification. As described in the next section, in this work we use support vector machines (SVM) for learning a model separating the two classes ‘live’ (*y* = 1) and ‘spoof’ (*y* = 0).

We remark that we considered a number of conceivable alternatives which include: (i) using the original, signed coefficients instead of their absolute value for the comparison in Eq (2), (ii) considering the leading sign of coefficients, i.e. relative frequencies that e.g. two coefficients are simultaneously positive, (iii) normalizing the rotation invariant image patches to a mean value of zero and a standard deviation of one before computing the discrete cosine transform. All these alternatives produce binary patterns which are also useful for classification. However, we have found that the afore described computation of the CCP descriptor has clearly the greatest discriminative power in the context of fingerprint liveness detection.

### 2.3 Classification

For classifying test set images, we compute the CCP feature vector in the way we have learned on the training set and we classify an input image as ‘live’ (*y* = 1) or ‘spoof’ (*y* = 0) by the trained SVM model.

### 2.4 Connection to Decision Trees and Fisher’s Linear Discriminant

The proposed criterion in Eq (1) at the learning stage shares commonalities with two well-known methods in machine learning and statistics: decision trees [43, 44] and the computation of Fisher’s linear discriminant (see Chapter 3 in [45]).

For constructing a decision tree, a standard approach is choose in each iteration the attribute which achieves the best split the set of training examples into separate sets. Here, “best” can be formalized by choosing a criterion like e.g. information gain [43]. Intuitively, it means that after the split each separate set tends to have clear majority and minority classes. In this analogy to decision trees, imagine that the criterion in Eq (1) simultaneously chooses the attribute for branching (indices *i* and *j* for the comparison), the splitting threshold value *δ* and it maximizes the differences between the two classes ‘live’ and ‘spoof’ for each branch.

For Fisher’s linear discriminant analysis, we assume that we are given a set of *n* *d*-dimensional samples *x*_1_, …, *x*_*n*_. Each sample point belongs to one of two classes and we consider the scalar dot project $y=w^{t}x$. Fisher’s criterion is defined as

$$\begin{array}{c} J\left(w\right)=\frac{{|{\tilde{m}}_{1}-{\tilde{m}}_{2}|}^{2}}{{\tilde{s}}_{1}^{2}+{\tilde{s}}_{2}^{2}}, \end{array}$$ (4)

where ${\tilde{m}}_{1}$ and ${\tilde{m}}_{2}$ are the sample means of the projected points and ${\tilde{s}}_{1}$ and ${\tilde{s}}_{2}$ are estimates of their standard deviations. Fisher’s linear discriminant enables an optimal choice of w which maximizes the expression in Eq (4), see Chapter 3, Section 3.8.2 in [45] for more details.

For the above described discriminant analysis *n* *d*-dimensional samples are already given as input, whereas in the situation of designing a binary pattern from scratch, each choice of a comparison defines one additional dimension. We can still shape our data based on their statistics on the whole training set, i.e. we can select the e.g. 8 most discriminative dimensions out of 6480 possibilities for *n* = 9. Fisher’s linear discriminant analysis maximizes the inter-class variability and minimizes intra-class variability. Here, with only two possible values arising from each comparison, we are interested only in maximizing the difference between the relative frequencies between the two classes as defined in Eq (1). As described in Section 2.2, we add the best comparison according to Eq (1) in each step. Therefore, Eq (1) maximizes the inter-class differences.

## 3 Experimental Results

**Fig 4:**
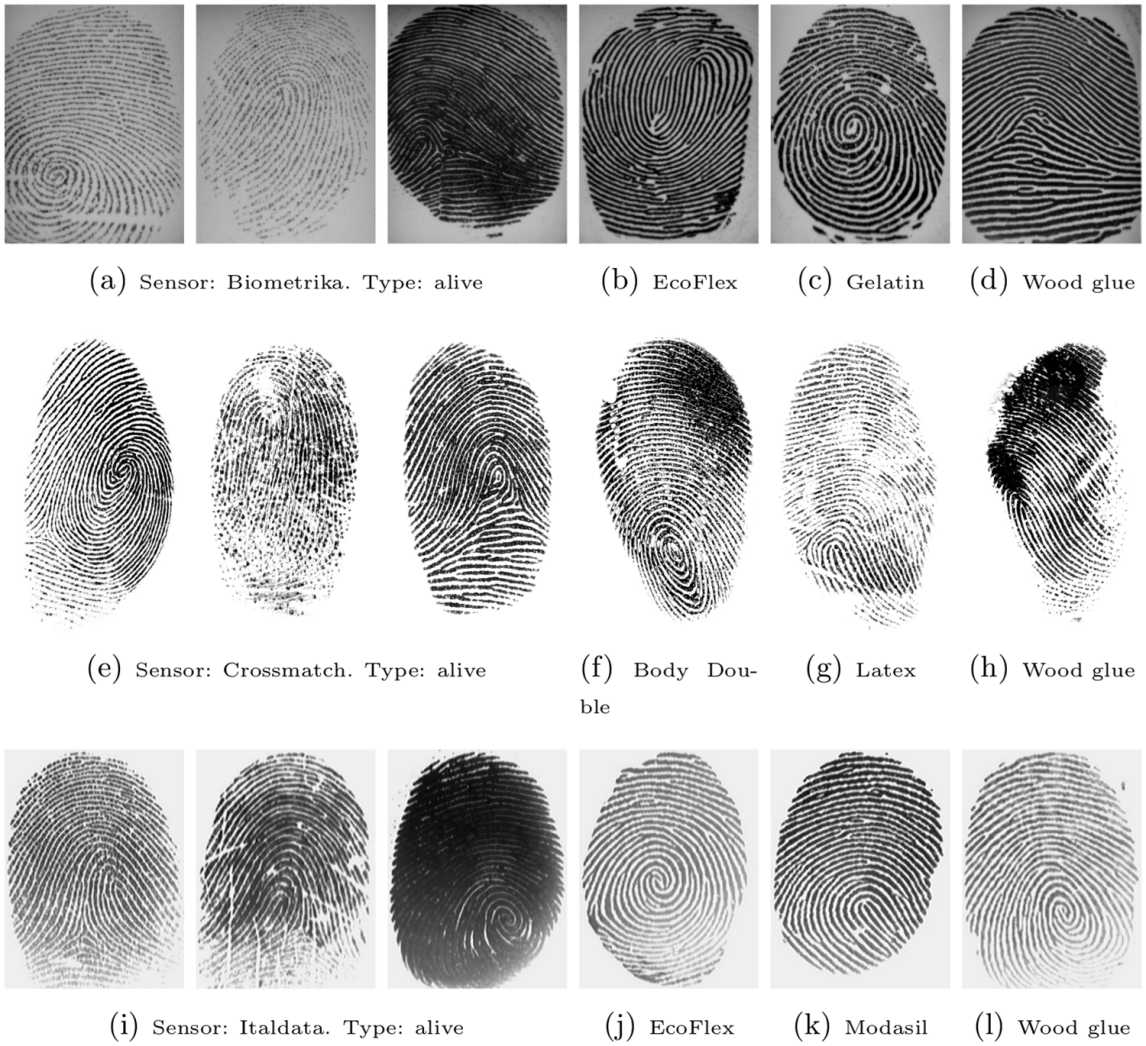
Example images from LivDet 2013 [42] after background removal by the FDB method [38]. The first row depicts images acquired on a Biometrika sensor, the second row from a Crossmatch sensor and the third row from an Italdata sensor. The leftmost three columns show images of real, alive fingers and rightmost three columns images of fake fingers (spoof material indicated in legend).

For comparability with results of the LivDet 2013 competition [42], we follow the identical evaluation protocol. SVMs with a linear kernel (*C* = 1.0) have been used for all methods and features have been rescaled to the range from −1 to 1. Experiments have been performed using LIBSVM [46] for the three databases acquired on optical sensors from Biometrika, Crossmatch and Italdata. Example images are depicted in Fig 4. The images of the swipe sensor database have a dimension of 208×1500 pixels and they would require sensor-specific, special preprocessing before basic steps like fingerprint segmentation and orientation field become possible. Therefore, the swipe sensor database has not been considered in these experiments.

The liveness detection accuracy reported in Table 1 has been computed as follows: $a=\frac{D}{N}$, where *D* is the number of correct decisions (classifying an image of an alive finger as ‘alive’ and classifying an image of a fake finger as ‘spoof’) and *N* is the number of all decisions. Further metrics considered in the literature are the normal presentation classification error rate (NPCER) which is the proportion of live fingerprints incorrectly classified as spoofs. This rate is called ‘FerrLive’ in [42]. Correspondingly, the attack presentation classification error rate (APCER) (or ‘FerrFake’ [42]) is the proportion of spoof fingerprints incorrectly classified as live. The half total error rate (HTER) is defined as $\text{HTER}=\frac{\text{NPCER+APCER}}{2}$. Please note that the accuracy *a* = 1−HTER, if there the number of live and spoof samples in the test set is identical which is the case for the Biometrika and Italdata databases listed in Table 1.

The comparison includes the best algorithm from LivDet 2013, a pore analysis based approach [47], histograms of invariant gradients (HIG) [37], Weber local descriptor (WLD) [10], local phase quantization (LPQ) [48] and local binary patterns (LBP) [6].

In 2008, LBP has been applied for liveness detection by Nikam and Agarwal [49] in form of rotation invariant uniform LBP which leads to a 54-dimensional feature vector. To improve the liveness detection performance, Ghiani *et al.*[50] proposed a fusion of LBP and LPQ by feature concatenation. WLD, LPQ and LBP, and combinations of LBP+LPQ and WLD+LPQ have been considered by Gragnaniello *et al.*[51]. These two feature combinations have also been evaluated for the LivDet 2013 databases as reported in Table 1. Combining elements from WLD and LPQ by considering their joint distribution (instead of feature concatenation) has been proposed in [52]. A Wavelet-Markov local descriptor has been suggested in [53].

In our implementation of the WLD descriptor [10], we used *α* = 3, *β* = 5, 120 bins for differential excitation and 8 orientation bins. Therefore, the dimension of the WLD vector is 8⋅120 = 960. We have implemented LBP as described in [49] which results in a feature vector of dimension 54. Our implementation of LPQ is denoted as modified LPQ (MLPQ) in Table 1. In comparison to the original LPQ, we have made the following modification: we consider the estimated orientation field and we compute rotation invariant image patches as described in Section 2. We perform the short time Fourier transform (STFT) for each rotation invariant image patch and compute the LPQ descriptor with a dimension of 2^8^ = 256. The implementation of the histograms of invariant gradients (HIG) [37] in Table 1 uses an orientation field estimation by the line sensor method [41] which has recently been adopted for detecting filament structures in microscopy images of human stem cells [54].

### 3.1 CCP Histogram Concatenation

We have evaluated four versions of CCP. As described in the previous section, a version with *b* = 8 comparisons and a histogram dimensionality of 2^8^ = 256. This CCP histogram comprises the joint distribution of *b* = 8 comparisons and is denoted as CCP 1 × 8 bit in Table 1.

Alternatively, we selected 24 comparisons which are grouped into four groups. The first *b* = 6 comparisons are used to compute the first histogram with 2^6^ = 64 bins. The next *b* = 6 comparisons (number 7 to 12 in the list from 1 to 24) are utilized to construct the second histogram with 64 bins. The third and fourth histogram are computed correspondingly from comparisons 13 to 18 and from comparisons 19 to 24. Finally, all four histograms with 64 bins are concatenated to form a feature vector with dimensionality 256. This feature vector is referred to as CCP 4 × 6 bit in Table 1.

Additionally, we have considered 16 comparisons sorted into two histograms (*b* = 8). By concatenation, we obtain a feature vector of length 2⋅2^8^ = 512. The fourth version involves eight times *b* = 6 comparisons. Hence, we concatenate 8 descriptors of length 2^6^ = 64 to a final feature vector of size 512.

### 3.2 Computational Complexity

A typical fingerprint recognition system (FRS) performs fingerprint segmentation [55], orientation field estimation, image enhancement, minutiae extraction and fingerprint matching [56–60]. Methods for fingerprint liveness detection and fingerprint alteration detection [61] can be considered as add-on modules to a FRS which aim to protect against these two types of presentation attacks [24]. The mean computational runtime for computing a CCP histogram with *n* = 9 pixels patch size and *b* = 8 comparisons is 0.7 seconds per image for the Biometrika database and 0.8 seconds per image for the Crossmatch and Italdata databases. for a not optimized Java implementation using one core of an Intel Core i7 CPU with 3.20 GHz. For a CCP descriptor of *b* = 8 comparisons, a rotationally invariant patch and 16 DCT coefficients are to be computed (or less than 16 if some coefficients appear in multiple comparisons). The computationally least expensive descriptor is LBP which involves 8 comparisons of two pixel values. The computation of the LPQ descriptor requires a short time Fourier transform for each image patch and its runtime depends on the patch size and efficiency of the Fourier transform implementation. Basically, all compared descriptors are suitable for real-time applications.

## 4 Conclusion

The experimental results reported in the previous section show that the proposed novel descriptor provides a very useful feature for fingerprint liveness detection. We intend to investigate the combination of several local descriptors for further improvements of the fingerprint liveness detection performance.

Possibilities for future work include the application of the proposed CCP descriptor in other areas like e.g. biological and medical image processing, texture recognition, face recognition and iris recognition, as well as liveness detection for face and iris images.

Moreover, fingerprint image compression is a topic which deserves further research. We plan to explore how the rotation invariant image patches can improve the DCT based compression in comparison to [21] and in comparison to the AM-FM based compression [29]. The required orientation field can be compressed to an extremely high degree. Existing methods for reconstructing an OF from a minutiae template face problems only in the area around singular points [28].
